# Dancing Bees Improve Colony Foraging Success as Long-Term Benefits Outweigh Short-Term Costs

**DOI:** 10.1371/journal.pone.0104660

**Published:** 2014-08-20

**Authors:** Roger Schürch, Christoph Grüter

**Affiliations:** 1 Laboratory of Apiculture and Social Insects, School of Life Sciences, University of Sussex, Falmer, United Kingdom; 2 Departamento de Biologia da Faculdade de Filosofia, Ciências e Letras de Ribeirão Preto, Universidade de São Paulo, Ribeirão Preto, São Paulo, Brazil; Universidade de São Paulo, Brazil

## Abstract

Waggle dancing bees provide nestmates with spatial information about high quality resources. Surprisingly, attempts to quantify the benefits of this encoded spatial information have failed to find positive effects on colony foraging success under many ecological circumstances. Experimental designs have often involved measuring the foraging success of colonies that were repeatedly switched between oriented dances *versus* disoriented dances (i.e. communicating vectors *versus* not communicating vectors). However, if recruited bees continue to visit profitable food sources for more than one day, this procedure would lead to confounded results because of the long-term effects of successful recruitment events. Using agent-based simulations, we found that spatial information was beneficial in almost all ecological situations. Contrary to common belief, the benefits of recruitment increased with environmental stability because benefits can accumulate over time to outweigh the short-term costs of recruitment. Furthermore, we found that in simulations mimicking previous experiments, the benefits of communication were considerably underestimated (at low food density) or not detected at all (at medium and high densities). Our results suggest that the benefits of waggle dance communication are currently underestimated and that different experimental designs, which account for potential long-term benefits, are needed to measure empirically how spatial information affects colony foraging success.

## Introduction

Colony success in social insects often depends on the colony's ability to mobilize workers and allocate them to where work is needed [1]-[4]. Accordingly, insects have evolved different ways to communicate in these situations, such as when nestmates must be recruited to valuable resources [1], [2]. One of the most remarkable means of recruitment is the honeybee waggle dance. Foragers perform this dance-like behavior inside the nest after finding a profitable food source or on a swarm during nest-hunting to advertise suitable nest-sites [3], [5]-[8]. Other foragers following a waggle dance learn the location and are subsequently able to fly to the area of the food source [8]–[12], where they use additional visual and olfactory information to localize the food [6], [8], [13], [14].

Only recently have attempts been made to quantify empirically the colony level benefits of this spatial information [15]–[19]. Surprisingly, these studies found that colonies would often not benefit from spatial communication [16]–[19]. For example, Donaldson-Matasci and Dornhaus [17] tested the effect of spatial communication in five different habitats, but found a positive effect of communication only in one. Dornhaus & Chittka [18] found benefits only in a tropical habitat, but not in temperate European habitats. These findings suggest that the benefits of spatial information strongly depend on the spatiotemporal distribution of food sources, a conclusion that is in agreement with theoretical studies [20]–[22]. Recruitment by waggle dances is costly in terms of time and energy [21], [23], [24] and it is thus conceivable that these costs outweigh the benefits under certain conditions, for example when food sources are easy to find by independent search (scouting).

However, the apparent absence of benefits resulting from spatial information use in many habitats could also be the result of confounding effects caused by the experimental designs used to quantify these benefits. In previous studies, researchers took advantage of the fact that honeybees are unable to perform oriented dances on a horizontal comb with no or only diffuse light [16]–[19]. It is thus possible to create colonies with oriented (with spatial information; SI) or disoriented (no direction information; NI) dances and compare the foraging success of colonies in these two conditions. Importantly, in these studies, colonies were kept in one condition for relatively short time periods (2 or 3 days in [16]–[18]; a variable number of days with a mean of ∼12 days in [19]) and repeatedly switched between the two experimental conditions (SI vs. NI), presumably to use paired statistical tests.

If a bee that was recruited on the last day of the SI treatment returns to that food source on the following day, the food she collects on that day will be added to the NI treatment, even though her success was a consequence of acquiring spatial information during the SI treatment. Thus, the continued availability of food sources combined with “site fidelity” (persistency) leads to confounded results and makes the interpretation of such data challenging. This seems particularly important since flower patches can remain in bloom for weeks or even months [25], and honeybees often return to the same foraging sites for days and up to 3 weeks [6], [8], [26]–[34].

Here we explored, using an agent-based simulation model, whether there is a benefit to the use of spatial information over longer periods of time than previously explored. We hypothesized that forager persistency causes long-term benefits of successful recruitment events, and therefore the benefits of spatial information might have been underestimated in experimental designs such as those used in empirical studies. Furthermore, we hypothesized that the degree to which results are confounded depends on various factors, including the duration of the experimental period, the longevity of food patches, the density of food sources, and the size of the colony.

## Model Description

The present model is a spatially explicit agent-based simulation model (ABM) of two types of agents in a foraging context, scouts and recruits. We used NetLogo 4.1.3 to implement the model (Text S1), and we follow the “Overview, Design Concepts, and Details” (ODD) protocol in our description of it below [35], [36].

### Purpose

We explored how potential long-term effects of spatial communication by means of waggle dances affect honeybee colony success in different environments. We then tested how different experimental designs that were used previously to measure empirically the benefits of spatial communication [16]–[19] affect foraging success of our virtual colonies in different environments. Our model shared many parameters with previous models, but was different in two key aspects. First, rather than exploring the effects of spatial communication during a few hours [20] or days [22], we monitored colonies during 48 days, allowing us to explore long-term effects of spatial communication. Second, recruits had a high probability of returning to profitable food sources on subsequent days (Figure S1). This assumption is well established by empirical research [29], [30], [33] – bees can even persistently visit unrewarding food sources up to 7 days [37].

### Entities, state variables and scale

Our agents were located on a two-dimensional square grid with 201×201 patches. The agents' nest was located on the center patch. Every other patch on the grid could either be void or contain suitable forage for our agents. Food patches were of variable quality (Table 1) and lasted for a limited number of days.

**Table 1:** Overview of the model parameters and the values used in our simulations.

		Default values	Other values tested
*N_Scouts_*	Number of scouts in the agent population	30	60, 120
*N_Recruits_*	Number of recruits in the agent population	270	540, 1080
*t_Day_*	Time steps per day	8640 time steps	
*t_Init_*	Time before experimental switches start	3 days	
*t_Switch_*	Duration of experimental switches	3 days	2, 12 days
*p_Exit_*	Probability of a scout to leave the nest per time step [39]	0.815	0.01, 0.1, 0.5, 0.8
*p_RS_*	Probability that idle recruits will leave the nest and scout per time step [22]	0.00009	
*d_Patch_*	Density of patches	0.05	0.01, 0.1
*a*_max_	Maximum age of patches	14 days	1, 7, 28
*y_Patch_*	Yield of a foraging trip [82]	50 mg	
*t_Patch_* ± σ*_t_*	Mean ± SD nectar handling time on patch [28], [83]	180 ± 60 time steps	
*t_Nest_*	Nectar handling time in the nest [84]	6 time steps	
*q_Patch_* ± σ*_q_*	Mean ± SD nectar quality [51]	1 ± 0.2 mol/l	
*v*	Agent speed	0.7 patch width/time step	
*µ_Lévi_*	Lévi flight parameter [48]	2.4	1, 2, 3, 4
*c_Rest_*	Energy costs per time step *t* when resting [85], [86]	0.04861 J	
*c_Move_*	Energy costs per time step *t* when moving [22], [85]	9 × *c*_Rest_ J	
*m*	Mortality per time step *t* [86]	0.000007	
*E*_mol_	Energy per mol sugar	5645000 J	
*c_Recruit_*	Energy costs incurred by recruits [22]	325 J	0 J
*N_Dance_*	Number of recruits following a dance of a scout	1	
*p_Dance_*	Probability of dance success [43], [44], [87]	0.25	

The population of agents (*N_default_*  =  300) consisted of two groups, the scouts (*N_default_*  =  30) and the recruits (*N_default_*  =  270). We used a default colony size of 300 agents, but we also tested other colony sizes to explore if results are sensitive to colony size. Colony size had no effect on the benefits of communication in a previous model [22] and was chosen to be similar to colony size in [38]. Honeybee scouts usually represent 5–25% of all foragers [3]. They search for food source locations without following waggle dances and have a high probability to abandon even rewarding food sources and search for new ones [39]. Scouts could assume any of six states: idle in the colony, scouting for food sources, feeding at a food source, returning to the colony, recruiting of idle foragers to the newly discovered food source, returning to the nest without having discovered food. Recruits decoded waggle dances to find a food patch, but they could also scout (Table 1) if they were unable to find a dancing bee [40]. Recruits could adopt any of 8 states: idle in the nest, idly waiting to be recruited, flying to food source, feeding on food source, returning to colony after feeding, recruiting new idle foragers, scouting for forage, returning to the colony without having discovered food.

Simulations were run in discrete time steps (*t*). We choose the model parameters (Table 1) such that one time step in a simulation corresponded to approximately 10 s real time, so that each simulated day was *t_day_*  =  8640 time steps long, with days starting at 6:00 in the morning. The simulated world allowed foraging when the following condition was true: 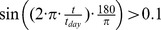. Conditions were therefore suitable between 6:23 and 17:37 real time. There was no change of foraging time with the progression of a simulation run (no seasonal change), and all days were assumed to have weather suitable for the agents to forage all day long. Often plants offer nectar during the better part of the day, but in varying amounts and qualities [41]. With our parameters (Table 1), the width of a patch corresponded to ca. 100 m, e.g. a group of trees or a flower field, and the world expanded 10 km from the nest patch. No foraging occured on the central patch. This means that our agents could not exploit food sources that were closer than 50 m to the nest. The agents could occupy any specific location on the two-dimensional plane (i.e. they were not restricted to move on a lattice) at every point in time.

### Process overview and scheduling

Each simulation experiment was preceded by a 3 day non-experimental phase where a colony of agents learned about the foraging patches surrounding the nest. Only then did the 48 days experimental phase start. Thus, in total each simulation run lasted 51 days, or 440,640 time steps.

At the beginning of each simulation run and every subsequent morning, idle scouts left the nest with a probability *p_Exit_*, and idle recruits waiting for food locations to be communicated to them (dances) left the nest with a probability *p_RS_* and started scouting. We chose *p_Exit_* relatively high, so that scouts left the hive early in the morning [39]. Successful scouts or scouting recruits fed on patches that offered forage, and then returned to the nest. After unloading, they could communicate the location of the food source in a ritualized dance to *N_Dance_* recruits, each of which had a probability of *p_Dance_* to learn the food source. *N_Dance_*  =  1 and *p_Dance_*  =  0.25 were chosen so that each dance had a 25% probability to recruit a forager to the advertised food patch (empirically determined values usually range from 0.1 to 1.0; [22], [23], [42]–[44]). The probability to dance *p_d_* depended on the quality of the visited foraging patch *q_Patch_* and the distance to this patch (Figure S1), as well as on the current influx of returned foragers (scouts *N_rs_* and recruits *N_rr,_* see Figure S2). A higher influx of foragers decreased the probability to dance [3], [45], [46]. On days when dances were disoriented (NI), dances animated recruits to scout for themselves instead, with *N_Dance_*  =  1 and *p_Dance_*  =  1, i.e., one recruit per dance left the hive. Scouts and recruits became idle once finished with dancing, from which state they could then leave the nest again with *p*_Exit_. Scouts consequently searched for new food sources, while recruits re-visited the previously visited food source for the rest of the day or until they died.

Every morning recruits with foraging experience decided whether to continue visiting the food source they had been visiting on the previous day or became idle recruits. The probability to abandon the food source depended on the quality of the forage, as well as on the distance to the patch (Figure S1), as did the probability to dance. If they did not abandon the patch, they left the nest with the probability *p_Exit_*. If the patch had vanished overnight, the recruits returned to the nest and became idle recruits.

Each time step, every agent had a probability of *m* to die. If an agent died, a completely equivalent new agent was born in the nest. This agent lacked foraging experience, and started out as an idle scout or recruit.

Each day forage patches with an age that exceeded *a*_max_ were replaced by empty patches. And empty patches could become forage patches with a probability *d_Patch_* and quality *q_Patch_*.

#### Experiments 1: benefits of SI with varying food density and experimental designs

In the first sets of experiments we tested the agents in an environment with high (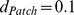), medium (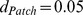) and low food density (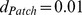). Analyzing the emergent foraging distances from model runs confirmed that these distributions led to naturally realistic median foraging distances between approx. 0.5–2 km [3], [47]. In each of the food densities, we performed model runs with colonies having either no switches, or 2-day, 3-day or 12-day cycles. In the no switch case, after the initial 3 days of information gathering, the colonies were allocated to their respective state (NI or SI) and did not switch state for the remaining 48 days. In the treatments with switches, we ran equal amounts of simulation runs with SI or NI in the first cycle, and subsequently switched back and forth.

#### Experiments 2: benefits of SI with varying patch longevities and experimental designs

In the second set of experiments we fixed the food patches at the medium density 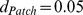. We ran simulations for patch longevity of a day (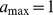), a week (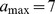), 2 weeks (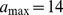) and 4 weeks (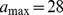). Like for the first set of experiments, we ran simulations with having either no switches between SI and NI states, or 2-day, 3-day or 12-day cycles.

### Design concepts

The model mimicked a central place foraging situation. Honeybee workers collected their food from a variety of sources, and brought it back to the nest for storage. Our model implemented the core properties necessary to simulate central place foraging with spatial information about the food source. The energy a colony of agents could extract from the simulated world was an emergent property. This energy intake could be affected by other emergent properties, such as waiting times during interactions. The agents adapted to their environment in many ways. Agents could sense the environment for whether it was good to leave the nest, they could assess whether a food source wass good enough for dancing, and whether a food source was worth visiting the next day. The agents could learn about the spatial location of the food source they had visited, or they could learn such a location from a returned forager, that communicated the location *via* oriented dances. This communication was the only interaction between agents. Whether a patch became a foraging patch was stochastically determined, as was the quality of the forage on the patch. Stochasticity also played a role in agents to determine whether agents left the nest, whether they danced for a food source, whether they could learn the location of a food source, whether they abandoned a food source, or whether they died.

At the end of each day, the nest stores of energy were sampled, as well as the median distance at which foraging occurred.

### Initialization

The patches, except the central nest patch, were randomly set to be foraging patches with a probability *d_Patch_*, and with quality *q_Patch_* ± σ*_qPatch_*. At initialization, the patch age was chosen to be uniformly distributed between 1 and *a*_max_. All scouts and recruits were initially set to be idle within the nest. The random number generator was initialized using the system's date-time.

### Submodels

#### The Lévy flight

To mimic the random search of honeybees, the agents moved using an optimal Lévy flight routine [48]. The agents first turned uniform randomly into a direction before moving. If the length of a leg was 0, because the journey on that leg had not started yet, a new distance for that leg was calculated as 
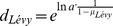
, where *a* was a uniform random variable drawn from the interval [0, 1) and a default *µ_Lévy_*  =  2.4 [48]. For the next time steps, the agent would move on the randomly chosen trajectory, until it had flown *d_Lévy_*.

#### The decision to dance or abandon food sources

Returned scouts and recruits could advertise the food source they had just visited with the dance, as long as the day still allowed for flying. For each time step, as long as the time spent unloading *t_Unloading_* < *t_Nest_*, the agent would decide whether to dance or not. We relied on Table 1 in [49] to determine the probability to dance for food sources of different energetic values at short foraging distances and on [50] to determine the relationship between the energetic value of a food source and the dancing probability at larger distances (up to several km, see Figure S1). Thus, the probability of dancing *p_d_* was calculated to be depending on distance *d* and the quality of the food source *q_Patch_*, 
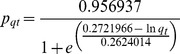
, where 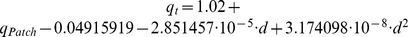. The probabilifty of dancing further depended on the influx of other foragers, 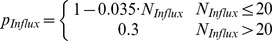,so that the final probability to dance was 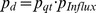 (see also Figure S2). Because recruits often need several excursions to find an advertised food source [23], [24], they incurred a penalty in terms of energy consumption 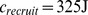 for locating the foraging patch in the field [22]. In case communication of the location was not allowed, recruits turned into scouting recruits without incurring the energy penalty.

Following Figure 6 in [51] we assumed that the shape of the curve of the relationship between food source profitability and dancing is the same as between food source profitability and abandonment of the food patch. The decision to abandon the patch was thus modelled analogously to the decision to dance or not. 
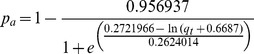
, where *q_t_* was calculated in exactly the same way as in the dance probability (Figure S1). The curve for the abandonment of the food source from one day to the next was approximated using data taken from [29], where a food source that led to a dance probability of 50% per foraging trip caused 80% of experienced foragers to return to this feeder the following day (Figure S1).

### Sensitivity analyses

Due to stochasticity we performed *N*  =  12 runs in each of our artificial experiments. We used standard errors (shown in Figures) to explore whether this number of runs was sufficient for interpretation without the need to perform additional statistical analysis.

To analyze how sensitive our model is to changes in parameters that were not under direct investigation, we chose default values presented in Table 1 for the duration of the experimental cycles *t_Switch_*, the food density *d_Patch_*, and the patch longevity *a*_max_. With these default values, we tested how the relative benefits of spatial information were influenced by colony size *N_Scouts_*+ *N_Recruits_*, the scouts' probability to exit the hive *p_Exit_*, the Lévi-flight parameter *µ_Lévi_*, and the energy costs of recruiting *c_Recruit_*. None of these factors alter the main results presented in that paper.

### Statistical methods

We used R to calculate means and standard errors per run or day [52]. In all cases, we ignored the 3 day acclimatization period when calculating means. To calculate means of the persistency of agents, we used the fitdistr function from the MASS package [53]. We assumed a Poisson distribution for the persistency.

## Results

### Experiments 1: switch durations, density and foraging success

**Figure 1:**
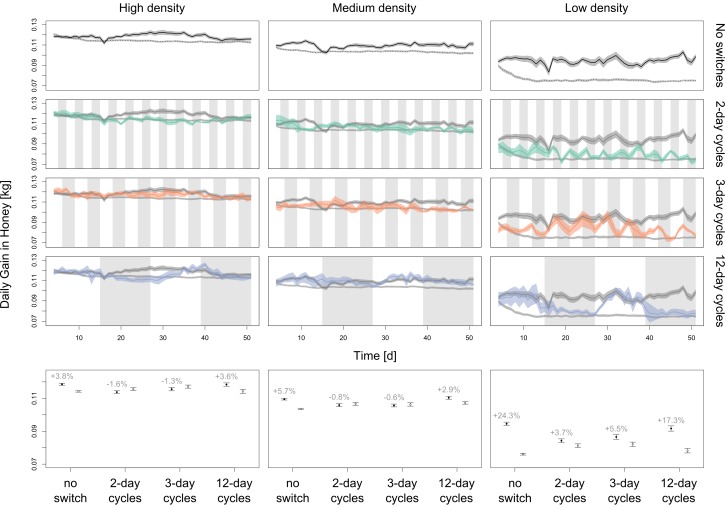
A comparison of foraging success measured as daily gain in honey under high, medium and low food densities, either with spatial information transfer (SI) or without spatial information transfer (NI). Model runs lasted 48 days after an initial period of 3 days SI (the 3 day acclimatization period is not shown). The first row shows the pure SI (solid black line) and NI (dotted black line) cases. These baselines are also shown for the model runs where switching between SI and NI occurred (rows 2–4, black solid and dotted lines). Rows 2–4 show the cases for SI (white background)/NI (grey background) cycles that last 2 days, 3 days or 12 days respectively. All the simulations shown here started with the SI treatment on day 4. The last row shows the aggregated simulation results (means ± s.e.) for the three food densities and SI/NI cycles (solid circles: SI, open circles: NI) including the second set of simulations that started with the NI treatment on day 4.

When there is no switching between SI and NI, spatial information transfer improves colony foraging success at all food source densities. The benefits increase when food resources are at lower densities (Figure 1; +2.5% at high density and +21.6% at low density). For all densities, the lack of spatial information leads to smaller variance in the colony energy intake compared to the situation where spatial information is always available (Figure 1).

In contrast, when switching colonies back and forth between SI and NI states every 2 or 3 days, the benefit of the information transfer remain undetected in all situations except when food density is low (Figure 1). But even in this situation, the detected relative benefits of SI are substantially lower than in untreated colonies with SI (+4.4% and +5.4% vs. + 21.6%). Of the 3 experimental designs, the results most similar to the untreated condition come from colonies subjected to 12-day experimental periods. Here, colonies benefit almost as much when having SI (+6.6% compared to NI over all densities), as in untreated colonies (+8.8%). The explanation for the lower relative benefits in switch experiments can be seen in Figure 1: switching between experimental states causes considerable carry-over effects, so that the collected energy on the first day of a new cycle is strongly dominated by the previous cycle.

In our model, the difference between recruits and scouts in individually collected energy per day is higher at low food densities but diminishes at higher densities. At low food densities (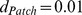) recruits have more than double the efficiency compared to scouts (236.1005 vs. 98.85329 ^J^/_day_ per forager) when information transfer is allowed. The increased efficiency then diminishes at higher densities (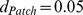, 243.8685 vs. 213.02885 ^J^/_day_ per forager; 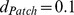, 250.1621 vs. 230.75986 ^J^/_day_ per forager).

### Experiments 2: switch durations, patch longevity and foraging success

**Figure 2:**
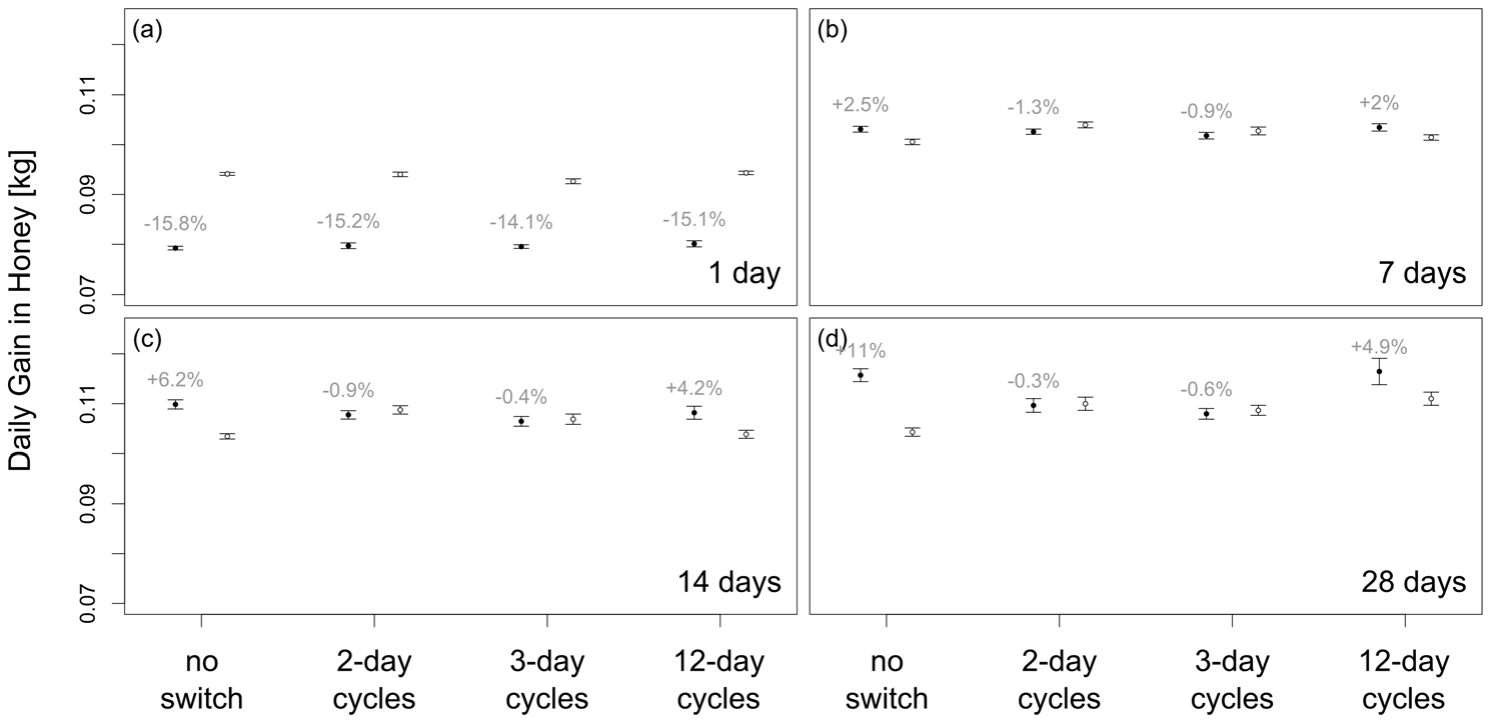
A comparison of foraging success measured as mean daily gain in honey (means ± s.e.) under varying patch longevities and with various intervals between experimental switches of spatial information transfer (SI) and no spatial information transfer (NI) treatments (no switches, 2-day cycles, 3-day cycles, 12-day cycles). The benefit of SI varies with patch longevity, with increasing benefits from one day (a), 7 days (b), 14 days (c), to 28 days (d). SI is more beneficial, if patches are relatively stable over time. Switching between SI and NI treatment hides the benefit of SI to a large degree (solid circle: SI, open circles: NI).

Experiment 1 used the default patch longevity of 14 days. Experiment 2 explored the effect of environmental stability by testing additional patch longevities at a medium food density. Surprisingly, the results show that the benefits of SI increase in untreated colonies as patch longevity increases. If patches only last 1 day, spatial dance information is even detrimental (−15.8%). But as patches last longer, colonies gain more from having spatial dance information (+11% for 28 days). Experiments with 2-day and 3-day experimental cycles provide very different results for all patch longevities, except for when patches last only for 1 day (Figure 2). If patches persist longer (≥ 7 days), colonies perform more or less equally well during SI and NI periods. Again, the best results are obtained using 12-day experimental cycles. But even in this situation the relative benefit of SI is less than half the benefit in the untreated colonies in a very stable environment (+11% vs. + 4.9%).

**Figure 3:**
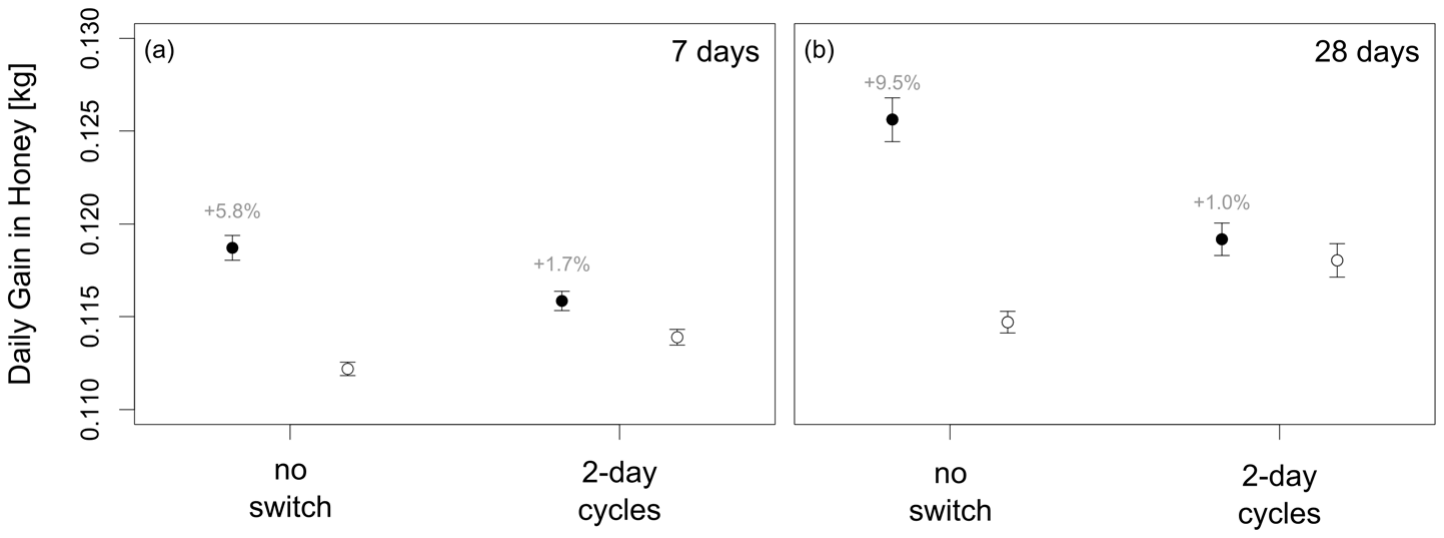
A comparison of foraging success measured as mean daily gain in honey (means ± s.e.) under the absence of costs to spatial information transfer (SI) and with SI, or no spatial information transfer (NI). The switching between SI and NI treatments masks the benefit of SI even if there are no costs to SI (solid circle: SI, open circles: NI).

Recruitment success and, consequently, the energetic costs of recruitment are likely to be variable [22]. To test whether this affects our findings, we tested a situation with higher recruitment success (*p_Dance_*  =  1) and no recruitment costs (*c_Recruit_*  =  0) in a separate set of model runs. This could represent a situation in certain tropical habitats with many large and clustered food patches, where recruits locate advertised foraging sites quickly. The simulations show that SI is again beneficial and that the relative benefits of SI increase in a more stable environment (Figure 3; +5.8% when patches last for 7 days, +9.5% when patches last for 21 days), but the benefits of SI are again underestimated by a factor of 3.5 to 9.5 for 7 day patches and 21 day patches respectively when using 2-day experimental cycles.

The longevity of food patches also has an impact on the individual foragers. The longer patches last, the longer single agents can be persistent. The mean persistency of agents ranged from 1.008±0.004 day when patches lasted for a day to 2.842±0.0124 days when patches lasted for 28 days (Figure S3), but the maximum persistence was only limited by the patch longevities.

### Sensitivity analyses

Qualitatively our main result, that switching hives between SI and NI treatments will obscure potential benefits of the dance, was robust under a wide range of parameters. The difference between SI and NI treatments were minimal for switching under a range of different colony sizes (Figure S4), differences in the random walk's Lévy parameter (Figure S5), or different probabilities for scouts to leave the hive (Figure S6).

## Discussion

Spatial location information of waggle dancing bees increased colony foraging success under almost all simulated circumstances. Spatial information did not improve colony success when patches lasted just 1 day, which is unrealistic for most natural habitats [25], [54]–[56]. The most surprising finding is that the relative benefits of spatial information increased as environmental stability increased (Figure 2). This contradicts the common assumption that dance information is most beneficial in an environment with ephemeral food sources [18], [19], [29], [57].

A closer examination of our model and the existing literature suggests that our results of long term benefits of the dance are plausible. Recruitment in honeybees is costly [21], [23], [24]: recruits need to wait for a dancing bee [21], and they usually require several field excursions before locating the advertised food source after following a dance [23], [58]–[60]. Thus, potential recruits incurred both energy and opportunity costs in our model. But after the advertised food source is located, the energetic returns are higher than for scouts. This result is consistent with empirical studies, which found that recruits tend to discover food sources of higher quality than scouts [23], [24], [60]. By dancing only for high quality food sources (see Figure S1 and [3], [8]) foraging bees effectively filter information, which allows recruits to exploit selectively the best food sources known to the colony [3], [61], [62]. After successful recruitment, costs of continuing to visit the food patch are low as bees quickly locate the previously visited patches using route-memory [63], [64], while benefits potentially remain high. The more stable the environment, the longer these benefits can accumulate. This is true even if there are short term fluctuations in a given patch's quality, such that it switches back and forth between profitable and non-profitable, because colonies with persistent individuals using private information can quickly decide which is the most profitable patch at a given time [65]. In contrast, if the environment completely changes (e.g., profitable patches become permanently unprofitable and vice versa), as in the case where patches only lasted for a day, recruits repeatedly have to pay the recruitment costs, while benefits remain short-lived.

One major difference between our model and previous models is our assumption that patches could last several days (and up to 4 weeks). Three lines of evidence suggest that this is a realistic assumption. First, it is known that flower patches in both tropical and temperate habitats often bloom for several days or even months [25], [54]–[56], [66]. Second, observations on naturally foraging honeybees show that foragers often return to the same patches for days and or even weeks [6], [8], [26], [28]–[30], [32]–[34]. Bees recruited by waggle dances are particularly likely to show site fidelity because food sources located by recruits are more profitable than those located by individually exploring bees [23], [24] and profitability positively affects site fidelity [33], [37]. In our model, foragers visited the same food patch for an average of 1 to 2.8 days, depending on the longevity of food patches (Figure S3). Third, honeybees readily learn the time of day when food sources are most rewarding and return to feeding sites on subsequent days at the appropriate time of day [27], [31], [67], [68]. This suggests that foragers are adapted to an environment where the spatiotemporal availability of the currently exploited food source is predictive for the next day. On the other hand, studies analyzing foraging locations by decoding waggle dances [3] show considerable daily changes in the locations that are advertised by dances. However, while such daily changes in dance maps inform us about the number of patches that receive increased exploitation (pp. 48 in [3]), they provide no information about how long individual foragers exploit patches. Clearly, the long-term foraging behavior of honeybee foragers in natural flower patches deserves further study.

Our findings help to explain the puzzling observation that experienced foragers following dances frequently ignore spatial waggle dance information [29], [69]–[74]. Our results show that foragers should continue to visit familiar food sources if these remain profitable in order to avoid recruitment costs and the lower benefits of individual exploration [23], [24], [60]. Decoding the spatial information of dances that advertise alternative food patches would become more beneficial if the currently visited patch deteriorates and using memory no longer provides rewards [29], [61], [75]. Accordingly, a recent simulation study found that individuals do best if they rely on learned behaviors most of the time and tailor social information-use to circumstances when the environment changes [76]. It is not yet entirely clear why experienced foragers follow dances at all if they subsequently ignore the spatial information. It is possible that these foragers follow dances mainly to acquire up-to-date information about the time period a particular flower species produces rewards, rather than its location, and is therefore worth inspecting by other foragers at other locations [6], [13], [60], [69], [77].

We hypothesized that treating colonies by repeatedly switching between SI and NI states leads to confounded results and obscures the benefits of spatial information. Our simulations corroborate this hypothesis. When we used short treatment periods (2-day or 3-day cycles), as in previous empirical studies [16]–[18], we often found no improved collection performance during periods with spatial information (Figure 1 and 2), even in environments where colonies with continuous access to SI (no switch) outperformed those without SI. The best estimates of dance benefits were obtained with 12-day treatment periods (similar to [19]). However, even this experimental design lead to a considerable underestimation of the relative benefits of spatial information in some environmental situations (Figure 1 and 2). Our simulations suggest that the problems of repeatedly switching between SI and NI are caused by carry-over effects (Figure 1): the foraging success of colonies during the first days of a new treatment is affected by the foraging success during the last days of the previous treatment (Figure 1). It takes a few days before these carry-over effects are no longer apparent. Allowing for site-fidelity, foragers collecting at a good food source have a high probability to continue visiting this food sources irrespective of treatment changes. Hence, colonies newly switched to the NI state will initially perform well because foragers continue to visit the high quality food sources to which they were recruited by waggle dances during SI periods. If we prevented the agents from being true to a site, these carry-over effects disappeared (Figure S7).

An additional problem for the interpretation of data from switch-experiments is that the degree to which data is confounded depends on the spatiotemporal distribution of food sources (Figure 1). This makes it especially challenging to meaningfully compare the foraging success of colonies in switch-experiments in different environments or seasons. To solve the methodological problems, we propose the following changes to experimental designs: First, switch experiments should allow colonies to recover for several days between SI and NI periods. Second, SI and NI periods should not be shorter than the average patch-age to make sure the environment changes substantially. With such a design, differences in the gained weight (or energy) should be noticeable between treatments. Alternatively, if only a qualitative result needs to be obtained (whether SI or NI is better), researchers could look at the change in weight gain over time instead of the weight gain. In other words, instead of recording the weight of colonies and calculating the day to day weight gain or loss ((weight on day t + 1) – (weight on day t)), one would calculate the day to day change in the weight gain or loss ((weight change on day t + 1) – (weight change on day t)). This latter solution does not involve a different experimental protocol and should therefore be straightforward to implement, but because of carry-over effects it would still be impossible to gain quantitative data on the difference between pure SI and NI treatments.

So far, we have discussed our results in the context of honeybee recruitment by waggle dances. However, the main findings of the model – the beneficial effects of social information, particularly in more stable environments – are probably not restricted to honeybees alone. If acquiring social information is (i) more costly and (ii) subsequently associated with higher rewards than asocial information (e.g. individual exploration), then we would expect the relative payoff of social information to increase with increasing environmental stability. In support of the first assumption (i), Dechaume-Moncharmont and colleagues [21] show with a model that social information is often costlier than asocial information due to time costs (waiting for a demonstrator). The recent finding that social information is usually of high quality because demonstrators perform the most successful behavior they know of [62] supports the second assumption (ii). However, empirical research is needed to estimate costs and benefits of different types of information and test the role of environmental stability.

Communication about food source locations is common in the Apini and many Meliponini (as pheromone trails), but not in bumble bees. The specific circumstances that lowered the costs or increased the benefits to dancing in *Apis* bees so it could evolve in the first place are not known, and our model is not concerned with the initial evolution of the dance. Recent phylogenies suggest that dancing evolved only once (reviewed in [5]), suggesting some constraints to dance evolution even if simpler forms of recruitment seem to evolve readily [78]. Temperate bumble bees might be exposed to different resource distribution [78]. Additionally, even though the range of tested colony sizes did not affect our main result, a critical colony size for the dance to be beneficial is likely: if the waiting costs to recruits are too long, for example because only a small work force is collecting food, the benefit of finding high quality food might not outweigh these waiting costs [79]–[81].

In summary, our study and previous simulation studies suggest that dancing is most beneficial in environments where food sources vary greatly in quality [20], are hard to find [22], [38] and persist for several days or weeks. In such an environment, spatial information helps a colony to allocate its foragers to highly profitable, but hard-to-find food sources and exploit those for extended time periods. We argue that dancing is beneficial in almost every natural environment, but new empirical studies using different experimental designs are needed to test this prediction.

## Supporting Information

Figure S1 The probability that an agent will dance upon its return to the colony, and the probability that a recruit will continue to visit a given food source was modelled to be dependent on the quality of the food as well as the distance to the food source. Depicted are the probabilities to dance in relation to food quality after foraging at 0, 2.5, 5 and 10 km (solid black lines), and the probability that a recruit will continue to visit a given food source the next day at 10 km foraging distance (grey line). At all times, the probability to visit a food source the next day is 30% higher at the food quality that elicits a dance response 50% of the times. See the main text for more details on implementation.
(TIF)

Figure S2 The probability of an agent to dance after a foraging trip in response to the influx of returned foragers. When more foragers return they are less likely to dance. We modelled this as a linear decrease of the probability to dance up to 20 foragers, at which level the dance probability remained constant.
(TIF)

Figure S3 The effect of patch longevity on foragers' persistence to remain with a foraging patch. The longer patches yield energy, the longer a forager stays with a patch to which it is committed (except for the patch longevity all parameters were set to the default values given in table 1 of the main text).
(TIF)

Figure S4 The effect of population size on the foraging success measured as mean daily gain in honey (means ± s.e.) with spatial information transfer (SI) or no spatial information transfer (NI). At all population sizes (**a)** *N*  =  300; **b)** *N*  =  600; **c)** *N*  =  1200) the switching between SI and NI treatments masks the benefit of SI (solid circle: SI, open circles: NI).
(TIF)

Figure S5 The effect of the Lévy flight parameter *µ*_Lévi_ on the foraging success measured as mean daily gain in honey (means ± s.e.) with spatial information transfer (SI) or no spatial information transfer (NI). At all levels for the Lévy flight parameter (**a)** *µ*_Lévi_  =  1; **b)** *µ*_Lévi_  =  2; **c)** *µ*_Lévi_  =  3; **d)** *µ*_Lévi_  =  4) the switching between SI and NI treatments masks the benefit of SI (solid circle: SI, open circles: NI).
(TIF)

Figure S6 The effect of the exit probability *p*_Exit_ on the foraging success measured as mean daily gain in honey (means ± s.e.) with spatial information transfer (SI) or no spatial information transfer (NI). At all exit probabilites (**a)** *p*_Exit_  =  1; **b)** *p*_Exit_  =  2; **c)** *p*_Exit_  =  3; **d)** *p*_Exit_  =  4) the switching between SI and NI treatments masks the benefit of SI (solid circle: SI, open circles: NI).
(TIF)

Figure S7 A comparison of foraging success measured as mean daily gain in honey (means ± s.e.) when recruits were switching food sources every day as opposed to visit food sources for multiple days (according to Fig. S1). Persistency allows the agents to gain long-term benefits from the spatial information transfer.
(TIF)

Text S1 The downloadable file, TextS1_BeeBenefitOfDance.nlogo, runs in NetLogo versions 4.1.3. NetLogo is freely available from the World Wide Web.
(NLOGO)
